# 4a-Hydroxy-9-(4-hydroxyphenyl)-4,4a,5,6,9,9a-hexahydro-3*H*-xanthene-1,8(2*H*,7*H*)-dione

**DOI:** 10.1107/S1600536811038335

**Published:** 2011-09-30

**Authors:** Liying Wang, Weicheng Lu, Yan Yang, Yulin Zhu

**Affiliations:** a School of Chemistry and Environment, South China Normal University, Guangzhou 510006, People’s Republic of China

## Abstract

The title compound, C_19_H_20_O_5_, was synthesized by the reaction of 1,3-cyclo­hexa­nedione and 4-hy­droxy­benzaldehyde in the presence of PdCl_2_ and thio­urea. The tetra­hydro­pyran ring and the six-membered cyclo­hexene ring adopt envelope conformations, and the six-membered cyclo­hexane ring is in a chair conformation. The crystal packing is stabilized by classical inter­molecular O—H⋯O hydrogen bonds and weak C—H⋯O inter­actions.

## Related literature

For applications of related compounds, see: Menchen *et al.* (2003 ▶); Saint-Ruf *et al.* (1972 ▶); Reddy *et al.* (2009 ▶); Mehdi *et al.* (2011 ▶). For the synthesis of related compounds, see: Karade *et al.* (2007 ▶); Luna *et al.* (2009 ▶). For related structures, see: Loh *et al.* (2011 ▶); Yang *et al.* (2011 ▶).
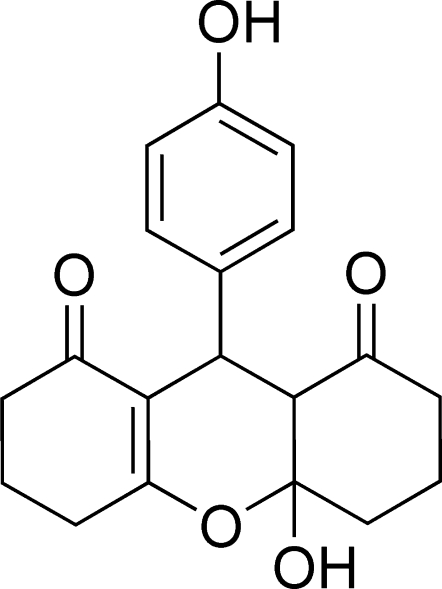


## Experimental

### 

#### Crystal data

C_19_H_20_O_5_
*M* *_r_* = 328.35
Monoclinic, 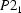

*a* = 9.014 (4) Å
*b* = 10.242 (4) Å
*c* = 9.289 (4) Å
β = 108.194 (4)°
*V* = 814.7 (6) Å^3^
*Z* = 2
Mo *K*α radiation
μ = 0.10 mm^−1^
*T* = 295 K
0.35 × 0.30 × 0.20 mm

#### Data collection

Bruker APEXII CCD diffractometer
Absorption correction: multi-scan (*SADABS*; Bruker, 2004 ▶) *T*_min_ = 0.967, *T*_max_ = 0.981
4840 measured reflections
1876 independent reflections
1652 reflections with *I* > 2σ(*I*)
*R*_int_ = 0.027

#### Refinement

*R*[*F* ^2^ > 2σ(*F* ^2^)] = 0.042
*wR*(*F* ^2^) = 0.113
*S* = 1.04
1863 reflections
219 parameters
14 restraints
H-atom parameters constrained
Δρ_max_ = 0.41 e Å^−3^
Δρ_min_ = −0.19 e Å^−3^

### 

Data collection: *APEX2* (Bruker, 2004 ▶); cell refinement: *SAINT* (Bruker, 2004 ▶); data reduction: *SAINT*; program(s) used to solve structure: *SHELXS97* (Sheldrick, 2008 ▶); program(s) used to refine structure: *SHELXL97* (Sheldrick, 2008 ▶); molecular graphics: *SHELXTL* (Sheldrick, 2008 ▶); software used to prepare material for publication: *SHELXTL*.

## Supplementary Material

Crystal structure: contains datablock(s) global, I. DOI: 10.1107/S1600536811038335/rk2296sup1.cif

Structure factors: contains datablock(s) I. DOI: 10.1107/S1600536811038335/rk2296Isup2.hkl

Supplementary material file. DOI: 10.1107/S1600536811038335/rk2296Isup3.cml

Additional supplementary materials:  crystallographic information; 3D view; checkCIF report

## Figures and Tables

**Table 1:** Hydrogen-bond geometry (Å, °)

*D*—H⋯*A*	*D*—H	H⋯*A*	*D*⋯*A*	*D*—H⋯*A*
O4—H4⋯O5^i^	0.82	2.07	2.852 (3)	160
O5—H5⋯O2^ii^	0.82	1.94	2.758 (4)	175
C4—H4*B*⋯O4^iii^	0.97	2.50	3.274 (5)	137
C8*A*—H13⋯O3^iv^	0.98	2.59	3.535 (4)	161
C12—H18⋯O2^ii^	0.93	2.55	3.251 (4)	132

Symmetry codes: (i) 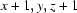; (ii) 
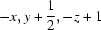
; (iii) 
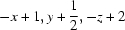
; (iv) 
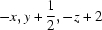
.

**Fig. 1.**
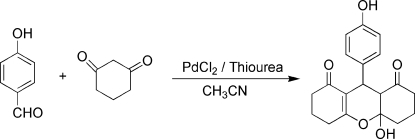
Palladium(II) chloride catalyzed synthesis of the title compound.

**Fig. 2.**
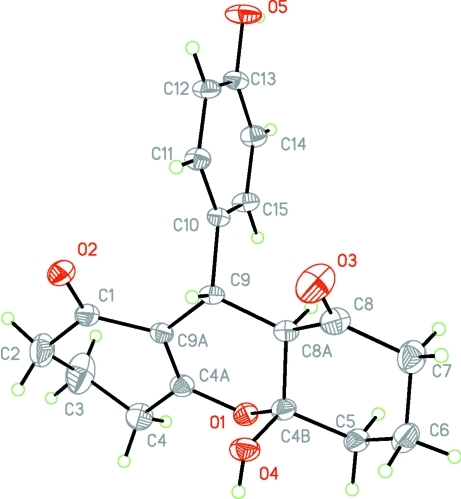
The molecular structure of title compound with the atom numbering scheme. Displacement ellipsoids are drawn at the 50% probability level. H atoms are presented as a small spheres of arbitrary radius.

## supplementary crystallographic information

### Comment

Xanthenes are an important class of heterocyclic compounds which attract researchers by their spectroscopic and biological properties. Their derivatives had been widely used as dyes, fluorescent materials for visualization and in laser technologies (Menchen *et al.*, 2003; Saint-Ruf *et al.*, 1972; Reddy *et al.*, 2009; Mehdi *et al.*, 2011). Due to their wide range of applications, a well established method used for the construction of xanthene unit was set up, which was a Tandem Michael reaction between 1,3-cyclohexanedione and benzaldehyde (Luna *et al.*, 2009; Karade *et al.*, 2007). The reaction between 1,3-cyclohexanedione and 4-hydroxybenzaldehyde in the presence of thiourea and PdCl_2_ proceeded to give the title compound in isolated yield 86% (Fig. 1).

The molecular structure of the title compound is illustrated in Fig. 2. There are no unusual bond lengths and angles in the molecule. The tricyclo system is connected with a phenly ring at the C9 position. The tetrahydropyran ring (O1/C4B/C8A/C9/C9A/C4A) and the six-membered cyclohexene ring (C1–C4/C4A/C9A) adopt envelope conformations, the other six-membered cyclohexane ring (C4B/C5–C8/C8A) is in a chair conformation. Other than the published structure 4*a*-hydroxy-9-(2-methoxyphenyl)-4,4*a*,5,6,7,8,9,9*a*-octahydro-3*H*-xanthene-1,8(2*H*)-dione or 3,4,4*a*,5,6,7,9,9*a*-octahydro-4*a*-hydroxyl-9-(4-chlorophenyl)-1*H*-xanthene-1,8(2*H*)-dione (Loh *et al.*, 2011; Yang *et al.*, 2011), the main structure of this compound is a derivated xanthene–dione fused tricyclo system with a hydroxyl group at its C4b position. The hydroxy group in phenly ring, tetrahydropyran ring with a hydroxyl group and carbonyl O atom allow the formation of two intermolecular O4—H4···O5^i^ and O5—H5···O2^ii^ hydrogen bonds. There are weak intermolecular C4—H4B···O4^iii^, C8A—H13···O3^iv^ and C12—H18···O2^ii^ interactions which link molecules into chains. Symmetyry codes: (i) *x*+1, *y*, *z*+1; (ii) -*x*, *y*+1/2, -*z*+1; (iii) -*x*+1, *y*+1/2, -*z*+2; (iv) -*x*, *y*+1/2, -*z*+2.

### Experimental

A mixture of 1,3-cyclohexanedione (1.12 g, 10 mmol), 4-hydroxybenzaldehyde (0.61 g, 5 mmol), thiourea (0.76 g,10 mmol) and PdCl_2_ (0.0020 mg) was refluxed in anhydrous acetonitrile (12 ml) at 373 K for 4 h. After being cooled to room temperature, the reaction mixture was poured into water. The white precipitate was filtered off with a silica pad, washed twice with anhydrous ethanol, and the filtrate was then dried under vacuum to yield the product in yield of 86%. Single crystals of the title compound were obtained by slow evaporation from anhydrous ethanol at room temperature to yield colourless, block-shaped crystal.

### Refinement

The H atoms were positioned geometrically and allowed to ride on their parent atoms, with C—H = 0.93–0.98Å and O—H = 0.82Å, respectively. The *U*_iso_ = 1.2*U*_eq_(C) and *U*_iso_ = 1.5*U*_eq_(O). 3350 Friedel pairs were merged during the refinement.

### Crystal data

**Table:**

C_19_H_20_O_5_	*F*(000) = 348.0
*M**_r_* = 328.35	*D*_x_ = 1.339 Mg m^−^^3^
Monoclinic, *P*2_1_	Mo *K*α radiation, λ = 0.71073 Å
Hall symbol: P 2yb	Cell parameters from 1885 reflections
*a* = 9.014 (4) Å	θ = 2.4–26.8°
*b* = 10.242 (4) Å	µ = 0.10 mm^−^^1^
*c* = 9.289 (4) Å	*T* = 295 K
β = 108.194 (4)°	Block, colourless
*V* = 814.7 (6) Å^3^	0.35 × 0.30 × 0.20 mm
*Z* = 2	

### Data collection

**Table:**

Bruker APEXII CCD diffractometer	1876 independent reflections
Radiation source: fine-focus sealed tube	1652 reflections with *I* > 2σ(*I*)
graphite	*R*_int_ = 0.027
φ– and ω–scans	θ_max_ = 27.0°, θ_min_ = 2.3°
Absorption correction: multi-scan (*SADABS*; Bruker, 2004)	*h* = −11→11
*T*_min_ = 0.967, *T*_max_ = 0.981	*k* = −13→12
4840 measured reflections	*l* = −10→11

### Refinement

**Table:**

Refinement on *F*^2^	Primary atom site location: structure-invariant direct methods
Least-squares matrix: full	Secondary atom site location: difference Fourier map
*R*[*F*^2^ > 2σ(*F*^2^)] = 0.042	Hydrogen site location: inferred from neighbouring sites
*wR*(*F*^2^) = 0.113	H-atom parameters constrained
*S* = 1.04	*w* = 1/[σ^2^(*F*_o_^2^) + (0.0658*P*)^2^ + 0.1291*P*] where *P* = (*F*_o_^2^ + 2*F*_c_^2^)/3
1863 reflections	(Δ/σ)_max_ = 0.001
219 parameters	Δρ_max_ = 0.41 e Å^−^^3^
14 restraints	Δρ_min_ = −0.19 e Å^−^^3^

### Special details

**Table:**

Geometry. All s.u.'s (except the s.u. in the dihedral angle between two l.s. planes) are estimated using the full covariance matrix. The cell s.u.'s are taken into account individually in the estimation of s.u.'s in distances, angles and torsion angles; correlations between s.u.'s in cell parameters are only used when they are defined by crystal symmetry. An approximate (isotropic) treatment of cell s.u.'s is used for estimating s.u.'s involving l.s. planes.
Refinement. Refinement of *F*^2^ against ALL reflections. The weighted *R*-factor w*R* and goodness of fit *S* are based on *F*^2^, conventional *R*-factors *R* are based on *F*, with *F* set to zero for negative *F*^2^. The threshold expression of *F*^2^ > 2σ(*F*^2^) is used only for calculating *R*-factors(gt) etc. and is not relevant to the choice of reflections for refinement. *R*-factors based on *F*^2^ are statistically about twice as large as those based on *F*, and *R*-factors based on ALL data will be even larger.

### Fractional atomic coordinates and isotropic or equivalent isotropic displacement parameters (Å^2^)

**Table:**

	*x*	*y*	*z*	*U*_iso_*/*U*_eq_	
C9	0.1903 (3)	0.1561 (2)	0.8768 (3)	0.0289 (5)	
H9	0.1954	0.0612	0.8912	0.035*	
C9A	0.3440 (3)	0.2024 (3)	0.8614 (3)	0.0307 (5)	
C4A	0.4337 (3)	0.2922 (3)	0.9542 (3)	0.0354 (6)	
C8A	0.1606 (3)	0.2192 (3)	1.0149 (3)	0.0307 (6)	
H13	0.1037	0.3003	0.9782	0.037*	
C5	0.2735 (3)	0.3317 (3)	1.2653 (3)	0.0407 (7)	
H5A	0.2174	0.4116	1.2264	0.049*	
H5B	0.3707	0.3551	1.3417	0.049*	
C8	0.0566 (3)	0.1394 (3)	1.0823 (3)	0.0432 (7)	
C6	0.1760 (4)	0.2471 (4)	1.3358 (4)	0.0483 (8)	
H6A	0.1504	0.2962	1.4142	0.058*	
H6B	0.2362	0.1712	1.3826	0.058*	
C1	0.4046 (3)	0.1347 (3)	0.7535 (3)	0.0375 (6)	
C4B	0.3076 (3)	0.2586 (3)	1.1383 (3)	0.0338 (6)	
C4	0.5792 (4)	0.3490 (4)	0.9360 (4)	0.0492 (8)	
H4A	0.6694	0.3118	1.0114	0.059*	
H4B	0.5803	0.4426	0.9525	0.059*	
C7	0.0251 (4)	0.2026 (4)	1.2164 (4)	0.0540 (9)	
H7A	−0.0287	0.1409	1.2619	0.065*	
H7B	−0.0428	0.2774	1.1820	0.065*	
C2	0.5553 (4)	0.1824 (4)	0.7353 (5)	0.0656 (11)	
H2A	0.6403	0.1290	0.7971	0.079*	
H2B	0.5513	0.1715	0.6304	0.079*	
C3	0.5892 (6)	0.3217 (5)	0.7787 (6)	0.0818 (15)	
H3A	0.5151	0.3768	0.7058	0.098*	
H3B	0.6930	0.3433	0.7760	0.098*	
O1	0.4005 (2)	0.34289 (19)	1.0760 (2)	0.0357 (4)	
O2	0.3391 (3)	0.0380 (2)	0.6848 (3)	0.0487 (6)	
O4	0.3938 (2)	0.1433 (2)	1.1914 (2)	0.0435 (5)	
H4	0.4795	0.1624	1.2506	0.065*	
O5	−0.3220 (3)	0.2708 (3)	0.3538 (3)	0.0548 (6)	
H5	−0.3326	0.3497	0.3398	0.082*	
O3	0.0004 (4)	0.0370 (3)	1.0282 (3)	0.0698 (8)	
C13	−0.1962 (3)	0.2473 (3)	0.4795 (3)	0.0340 (6)	
C11	0.0184 (3)	0.3158 (3)	0.6905 (3)	0.0350 (6)	
H19	0.0782	0.3830	0.7472	0.042*	
C10	0.0564 (3)	0.1873 (3)	0.7339 (3)	0.0275 (5)	
C14	−0.1588 (3)	0.1177 (3)	0.5195 (3)	0.0376 (6)	
H16	−0.2179	0.0507	0.4616	0.045*	
C15	−0.0334 (3)	0.0891 (3)	0.6455 (3)	0.0323 (6)	
H15	−0.0086	0.0024	0.6717	0.039*	
C12	−0.1073 (3)	0.3459 (3)	0.5641 (3)	0.0365 (6)	
H18	−0.1312	0.4325	0.5367	0.044*	

### Atomic displacement parameters (Å^2^)

**Table:**

	*U*^11^	*U*^22^	*U*^33^	*U*^12^	*U*^13^	*U*^23^
C9	0.0264 (11)	0.0269 (13)	0.0275 (13)	−0.0013 (10)	0.0000 (10)	0.0017 (10)
C9A	0.0270 (11)	0.0307 (13)	0.0306 (13)	0.0014 (10)	0.0034 (10)	0.0007 (11)
C4A	0.0330 (12)	0.0342 (14)	0.0358 (14)	−0.0032 (11)	0.0062 (11)	−0.0044 (12)
C8A	0.0306 (12)	0.0304 (14)	0.0278 (13)	−0.0016 (10)	0.0045 (10)	0.0013 (11)
C5	0.0407 (14)	0.0428 (17)	0.0348 (14)	0.0025 (13)	0.0064 (11)	−0.0078 (13)
C8	0.0421 (14)	0.0477 (17)	0.0379 (15)	−0.0097 (14)	0.0099 (12)	0.0021 (14)
C6	0.0621 (19)	0.0514 (19)	0.0340 (15)	0.0075 (16)	0.0186 (14)	0.0012 (14)
C1	0.0362 (13)	0.0389 (15)	0.0330 (14)	0.0042 (12)	0.0044 (11)	−0.0019 (12)
C4B	0.0330 (13)	0.0341 (14)	0.0301 (14)	−0.0014 (11)	0.0038 (11)	−0.0037 (11)
C4	0.0409 (15)	0.0504 (18)	0.0570 (19)	−0.0156 (14)	0.0165 (14)	−0.0104 (16)
C7	0.0548 (19)	0.061 (2)	0.053 (2)	−0.0116 (17)	0.0268 (16)	−0.0054 (18)
C2	0.0526 (19)	0.079 (3)	0.075 (3)	−0.0129 (19)	0.0353 (19)	−0.029 (2)
C3	0.089 (3)	0.077 (3)	0.105 (3)	−0.036 (3)	0.066 (3)	−0.027 (3)
O1	0.0346 (9)	0.0329 (10)	0.0368 (10)	−0.0065 (8)	0.0074 (8)	−0.0081 (8)
O2	0.0484 (12)	0.0473 (13)	0.0444 (13)	0.0007 (10)	0.0059 (10)	−0.0167 (10)
O4	0.0444 (11)	0.0390 (11)	0.0378 (11)	0.0083 (9)	−0.0005 (8)	0.0008 (9)
O5	0.0443 (11)	0.0498 (14)	0.0473 (13)	0.0012 (10)	−0.0190 (9)	0.0032 (11)
O3	0.0865 (18)	0.0670 (18)	0.0645 (18)	−0.0429 (15)	0.0359 (15)	−0.0146 (14)
C13	0.0264 (12)	0.0407 (15)	0.0285 (13)	0.0015 (11)	−0.0008 (10)	0.0030 (11)
C11	0.0354 (13)	0.0309 (14)	0.0306 (13)	−0.0029 (11)	−0.0014 (10)	0.0005 (11)
C10	0.0235 (11)	0.0307 (13)	0.0250 (12)	−0.0002 (10)	0.0031 (9)	0.0025 (10)
C14	0.0300 (12)	0.0396 (16)	0.0351 (14)	−0.0079 (11)	−0.0017 (11)	−0.0065 (12)
C15	0.0319 (12)	0.0268 (13)	0.0345 (14)	−0.0013 (10)	0.0050 (11)	0.0004 (11)
C12	0.0384 (13)	0.0300 (14)	0.0344 (14)	0.0038 (11)	0.0017 (11)	0.0051 (11)

### Geometric parameters (Å, °)

**Table:**

C9—C9A	1.513 (3)	C4—C3	1.518 (6)
C9—C10	1.523 (3)	C4—H4A	0.9700
C9—C8A	1.533 (4)	C4—H4B	0.9700
C9—H9	0.9800	C7—H7A	0.9700
C9A—C4A	1.345 (4)	C7—H7B	0.9700
C9A—C1	1.458 (4)	C2—C3	1.487 (6)
C4A—O1	1.360 (3)	C2—H2A	0.9700
C4A—C4	1.492 (4)	C2—H2B	0.9700
C8A—C4B	1.512 (3)	C3—H3A	0.9700
C8A—C8	1.518 (4)	C3—H3B	0.9700
C8A—H13	0.9800	O4—H4	0.8200
C5—C4B	1.508 (4)	O5—C13	1.371 (3)
C5—C6	1.521 (5)	O5—H5	0.8200
C5—H5A	0.9700	C13—C12	1.373 (4)
C5—H5B	0.9700	C13—C14	1.391 (4)
C8—O3	1.204 (4)	C11—C12	1.388 (4)
C8—C7	1.507 (5)	C11—C10	1.388 (4)
C6—C7	1.532 (5)	C11—H19	0.9300
C6—H6A	0.9700	C10—C15	1.387 (4)
C6—H6B	0.9700	C14—C15	1.381 (4)
C1—O2	1.225 (4)	C14—H16	0.9300
C1—C2	1.502 (5)	C15—H15	0.9300
C4B—O4	1.416 (4)	C12—H18	0.9300
C4B—O1	1.443 (3)		
			
C9A—C9—C10	110.6 (2)	C3—C4—H4A	109.5
C9A—C9—C8A	110.6 (2)	C4A—C4—H4B	109.5
C10—C9—C8A	110.0 (2)	C3—C4—H4B	109.5
C9A—C9—H9	108.5	H4A—C4—H4B	108.1
C10—C9—H9	108.5	C8—C7—C6	111.9 (3)
C8A—C9—H9	108.5	C8—C7—H7A	109.2
C4A—C9A—C1	119.1 (2)	C6—C7—H7A	109.2
C4A—C9A—C9	122.6 (2)	C8—C7—H7B	109.2
C1—C9A—C9	117.8 (2)	C6—C7—H7B	109.2
C9A—C4A—O1	123.3 (2)	H7A—C7—H7B	107.9
C9A—C4A—C4	124.6 (3)	C3—C2—C1	113.4 (3)
O1—C4A—C4	112.0 (2)	C3—C2—H2A	108.9
C4B—C8A—C8	109.8 (2)	C1—C2—H2A	108.9
C4B—C8A—C9	114.0 (2)	C3—C2—H2B	108.9
C8—C8A—C9	114.2 (2)	C1—C2—H2B	108.9
C4B—C8A—H13	106.0	H2A—C2—H2B	107.7
C8—C8A—H13	106.0	C2—C3—C4	111.7 (4)
C9—C8A—H13	106.0	C2—C3—H3A	109.3
C4B—C5—C6	109.9 (3)	C4—C3—H3A	109.3
C4B—C5—H5A	109.7	C2—C3—H3B	109.3
C6—C5—H5A	109.7	C4—C3—H3B	109.3
C4B—C5—H5B	109.7	H3A—C3—H3B	107.9
C6—C5—H5B	109.7	C4A—O1—C4B	114.3 (2)
H5A—C5—H5B	108.2	C4B—O4—H4	109.5
O3—C8—C7	123.7 (3)	C13—O5—H5	109.5
O3—C8—C8A	122.0 (3)	O5—C13—C12	122.5 (3)
C7—C8—C8A	114.2 (3)	O5—C13—C14	117.5 (2)
C5—C6—C7	111.1 (3)	C12—C13—C14	120.0 (2)
C5—C6—H6A	109.4	C12—C11—C10	121.2 (3)
C7—C6—H6A	109.4	C12—C11—H19	119.4
C5—C6—H6B	109.4	C10—C11—H19	119.4
C7—C6—H6B	109.4	C15—C10—C11	118.0 (2)
H6A—C6—H6B	108.0	C15—C10—C9	121.4 (2)
O2—C1—C9A	121.3 (3)	C11—C10—C9	120.6 (2)
O2—C1—C2	120.7 (3)	C15—C14—C13	119.6 (3)
C9A—C1—C2	117.9 (3)	C15—C14—H16	120.2
O4—C4B—O1	108.4 (2)	C13—C14—H16	120.2
O4—C4B—C5	111.4 (2)	C14—C15—C10	121.3 (3)
O1—C4B—C5	107.6 (2)	C14—C15—H15	119.3
O4—C4B—C8A	107.3 (2)	C10—C15—H15	119.3
O1—C4B—C8A	109.7 (2)	C13—C12—C11	119.8 (3)
C5—C4B—C8A	112.4 (2)	C13—C12—H18	120.1
C4A—C4—C3	110.8 (3)	C11—C12—H18	120.1
C4A—C4—H4A	109.5		
			
C10—C9—C9A—C4A	118.8 (3)	C9—C8A—C4B—C5	174.6 (2)
C8A—C9—C9A—C4A	−3.4 (3)	C9A—C4A—C4—C3	15.5 (5)
C10—C9—C9A—C1	−68.9 (3)	O1—C4A—C4—C3	−163.9 (3)
C8A—C9—C9A—C1	169.0 (2)	O3—C8—C7—C6	132.1 (4)
C1—C9A—C4A—O1	−167.6 (3)	C8A—C8—C7—C6	−50.7 (4)
C9—C9A—C4A—O1	4.7 (4)	C5—C6—C7—C8	52.4 (4)
C1—C9A—C4A—C4	13.1 (4)	O2—C1—C2—C3	159.2 (4)
C9—C9A—C4A—C4	−174.7 (3)	C9A—C1—C2—C3	−24.1 (5)
C9A—C9—C8A—C4B	−26.3 (3)	C1—C2—C3—C4	52.2 (6)
C10—C9—C8A—C4B	−148.8 (2)	C4A—C4—C3—C2	−47.4 (5)
C9A—C9—C8A—C8	−153.6 (2)	C9A—C4A—O1—C4B	25.7 (4)
C10—C9—C8A—C8	83.9 (3)	C4—C4A—O1—C4B	−154.9 (2)
C4B—C8A—C8—O3	−131.1 (3)	O4—C4B—O1—C4A	62.6 (3)
C9—C8A—C8—O3	−1.7 (4)	C5—C4B—O1—C4A	−176.7 (2)
C4B—C8A—C8—C7	51.6 (3)	C8A—C4B—O1—C4A	−54.2 (3)
C9—C8A—C8—C7	−179.0 (3)	C12—C11—C10—C15	1.1 (4)
C4B—C5—C6—C7	−56.4 (4)	C12—C11—C10—C9	−177.3 (2)
C4A—C9A—C1—O2	167.8 (3)	C9A—C9—C10—C15	117.6 (3)
C9—C9A—C1—O2	−4.8 (4)	C8A—C9—C10—C15	−120.0 (2)
C4A—C9A—C1—C2	−8.8 (4)	C9A—C9—C10—C11	−64.0 (3)
C9—C9A—C1—C2	178.6 (3)	C8A—C9—C10—C11	58.4 (3)
C6—C5—C4B—O4	−61.1 (3)	O5—C13—C14—C15	−179.8 (3)
C6—C5—C4B—O1	−179.8 (2)	C12—C13—C14—C15	0.9 (4)
C6—C5—C4B—C8A	59.3 (3)	C13—C14—C15—C10	0.2 (4)
C8—C8A—C4B—O4	67.0 (3)	C11—C10—C15—C14	−1.1 (4)
C9—C8A—C4B—O4	−62.6 (3)	C9—C10—C15—C14	177.3 (2)
C8—C8A—C4B—O1	−175.5 (2)	O5—C13—C12—C11	179.8 (3)
C9—C8A—C4B—O1	54.9 (3)	C14—C13—C12—C11	−0.9 (4)
C8—C8A—C4B—C5	−55.9 (3)	C10—C11—C12—C13	−0.1 (4)

### Hydrogen-bond geometry (Å, °)

**Table:**

*D*—H···*A*	*D*—H	H···*A*	*D*···*A*	*D*—H···*A*
O4—H4···O5^i^	0.82	2.07	2.852 (3)	160.
O5—H5···O2^ii^	0.82	1.94	2.758 (4)	175.
C4—H4B···O4^iii^	0.97	2.50	3.274 (5)	137.
C8A—H13···O3^iv^	0.98	2.59	3.535 (4)	161.
C12—H18···O2^ii^	0.93	2.55	3.251 (4)	132.

Symmetry codes: (i) *x*+1, *y*, *z*+1; (ii) −*x*, *y*+1/2, −*z*+1; (iii) −*x*+1, *y*+1/2, −*z*+2; (iv) −*x*, *y*+1/2, −*z*+2.
